# Bioactivity of PEGylated Graphene Oxide Nanoparticles Combined with Near-Infrared Laser Irradiation Studied in Colorectal Carcinoma Cells

**DOI:** 10.3390/nano11113061

**Published:** 2021-11-14

**Authors:** Natalia Krasteva, Dessislava Staneva, Bela Vasileva, George Miloshev, Milena Georgieva

**Affiliations:** 1 Institute of Biophysics and Biomedical Engineering, Bulgarian Academy of Sciences, “Acad. Georgi Bonchev” Str., Bl. 21, 1113 Sofia, Bulgaria; 2 Institute of Molecular Biology “Acad. R. Tsanev”, Bulgarian Academy of Sciences, “Acad. Georgi Bonchev” Str., Bl. 21, 1113 Sofia, Bulgaria; dessysta@gmail.com (D.S.); belavas@outlook.com (B.V.); karamolbiol@gmail.com (G.M.)

**Keywords:** graphene oxide (GO), GO–PEG, nanocarrier, bioactivity, near-infrared (NIR) light, photothermal therapy (PTT), colorectal carcinoma, gene expression, cell cycle, genotoxicity, mitotoxicity

## Abstract

Central focus in modern anticancer nanosystems is given to certain types of nanomaterials such as graphene oxide (GO). Its functionalization with polyethylene glycol (PEG) demonstrates high delivery efficiency and controllable release of proteins, bioimaging agents, chemotherapeutics and anticancer drugs. GO–PEG has a good biological safety profile, exhibits high NIR absorbance and capacity in photothermal treatment. To investigate the bioactivity of PEGylated GO NPs in combination with NIR irradiation on colorectal cancer cells we conducted experiments that aim to reveal the molecular mechanisms of action of this nanocarrier, combined with near-infrared light (NIR) on the high invasive Colon26 and the low invasive HT29 colon cancer cell lines. During reaching cancer cells the phototoxicity of GO–PEG is modulated by NIR laser irradiation. We observed that PEGylation of GO nanoparticles has well-pronounced biocompatibility toward colorectal carcinoma cells, besides their different malignant potential and treatment times. This biocompatibility is potentiated when GO–PEG treatment is combined with NIR irradiation, especially for cells cultured and treated for 24 h. The tested bioactivity of GO–PEG in combination with NIR irradiation induced little to no damages in DNA and did not influence the mitochondrial activity. Our findings demonstrate the potential of GO–PEG-based photoactivity as a nanosystem for colorectal cancer treatment.

## 1. Introduction

Despite immense efforts and billions of dollars invested each year in the search for new anticancer therapies, cancer continues to be the major lethality cause worldwide. One of the most malignant and deadly diseases occurring in elderly people is colorectal cancer (CRC). In 2020, CRC accounted for 10% of global cancer incidence and 9.4% of all cancer deaths which made it the third most common and the second deadliest tumor globally [1,2].

Currently, the conventional treatments for CRC include surgery, chemo- and radiotherapy and the choice depends mainly on the tumor stage. Surgery is operated for the early, localized stage while chemotherapy and radiotherapy are the main treatment for the advanced CRC stages [3]. All treatments, however, are accompanied by severe side effects and unsatisfactory results for cancer patients [4]. Poor tumor site-specificity, healthy tissue toxicity, and high tumor drug resistance are the main limitations of current therapies, thus decreasing the overall anticancer effectiveness [5]. Therefore, an urgent need for the development of novel strategies that overcome the limitations of conventional anticancer approaches exists. Gene therapy, immunotherapy, photodynamic and photothermal therapy are new and promising anticancer treatments but yet undiscovered expansively [4,6,7,8]. Among them, photothermal therapy (PTT) is a non-invasive approach with better patient outcomes than chemotherapy, especially in the treatment of drug-resistant tumors [9]. In near-infra red (NIR)-PTT the most important component of PTT—the exogenous phototherapeutic agents (photosensitizers, PSs)—are activated under appropriate NIR laser irradiation converting light into heat thus increasing the temperature in the cells, consequently triggering cell death [4]. PTT has many advantages among which are the following: (1) cancer cells have no good heat resistance; (2) the laser is an ideal external stimulus, which is easily regulated, focused, and remotely controlled, enabling more selective cancer targeting and elimination as well as minimized damage in the surrounding healthy tissues [4,10]; (3) NIR light induces mild hyperthermia that increases vascular permeability in tumor tissues for anticancer drugs [11,12]; (4) NIR light is absorbed by endogenous absorbers in tissues, which offers deeper tissue penetration in vivo [10,13,14]. The main disadvantage of phototherapy, however, lies in its adverse effects on the surrounding healthy tissues [15]. Extensive efforts are put into the design and development of highly efficient PSs [16]. An ideal photosensitizer should have the capacity to recognize tumor cells, target key organelles, and possess low toxicity during drug delivery into cancer cells with increased toxicity during irradiation [4,16]. Thus the required drug dose can be decreased and the cancer cell-killing efficiency can be enhanced with minimal side effects [16]. Recently, a simple and sensitive two-photon imaging fluorescent nitrogen-doped carbon dots (N-CDs) platform was constructed [17]. The aim was to detect β-glucuronidase as a tumor-invasive biomarker based on inner filter effect, to visually monitor anticancer drug loading by fluorescence resonance energy transfer (FRET), and to effectively treat cancer by chemotherapy.

For the past several years, mitochondria have attracted attention as a potential target for anticancer drugs. These organelles are highly sensitive to hyperthermia, which often results in mitochondrial damages and eventually to cell death [18]. In the light of modern anticancer therapies involving photothermal induction of cancer cells’ damage, the selective generation of hyperthermia by NIR-PTT in the mitochondria is expected to considerably improve therapeutic efficacy. Consequently, promises are given to anticancer treatments that combine hyperthermia and suppress ATP production via ROS-mediated mitochondrial dysfunction [19]. Several mitochondria-targeting compounds that cause a mitochondrial malfunction in tumor cells were proposed such as mitochondria-directed conventional drugs, mitochondrial protein-inhibiting agents, and mitochondria-targeted PSs [20]. PSs localize into various organelles within cells including the mitochondria, lysosomes, Golgi apparatus, and endoplasmic reticulum [16,21]. It is generally accepted that the site of subcellular localization of PSs is the main site of photodamage [22]. The phototoxic effect of PSs in cancer cells is governed by their photophysical properties [23]. Hence, the main aim of novel photothermal anticancer therapies involves the design of nanosystems that switch phototoxicity during drug delivery, thus ameliorating the PTT treatment and eliminating its side effects [16,24]. To design such nanosystems, both organic and inorganic nanostructured materials, were considered [25].

The central focus is given to certain types of nanomaterials such as graphene oxide (GO). The interest in GO is immense as it has a robust NIR absorption generating sufficient heat under NIR irradiation (when is “on”) and easily leads to cell destruction [26]. The one-atom thin sheet structure of graphene oxide makes it highly compatible with modern anticancer therapies especially with its high reactivity mainly due to the high content of oxygen-containing functional groups [27]. The advantages of GO as a promising nanocarrier of therapeutic drugs come from its high specific surface area with delocalized electrons allowing successful drug loading [28,29]. Moreover, GO oxygen functional groups can be easily modified for biomolecules attachment, which further improves its drug loading/delivery capacity [30,31]. GO functionalization with polyethylene glycol (PEG) demonstrates high delivery efficiency and controllable release of proteins, bio imaging agents, chemotherapeutics, and anticancer drugs. GO–PEG has a good biological safety profile [18,32,33,34] and exhibits high NIR absorbance and capacity in photothermal treatment [35]. Recently, we studied the physicochemical characteristics of PEGylated GO and introduced GO–PEG in combination with NIR irradiation as a biocompatible smart nanocarrier in colon cancer cells with enhanced physicochemical properties and higher biological compatibility [36]. In another study, we further expanded these experiments and demonstrated that this modification of GO leads to increased biocompatibility of GO–PEG for human blood cells too [37].

Here, we discuss our recent results on the bioactivity of PEGylated GO NPs in combination with NIR irradiation on colorectal cancer cells. We conducted experiments that aim to reveal the molecular mechanisms of action of this nanocarrier combined with near-infrared light (NIR) on the high invasive Colon26 and the low invasive HT29 colon cancer cell lines. During reaching the cancer cells the phototoxicity of GO–PEG is modulated by NIR laser irradiation. We investigated the cyto-, geno- and mitotoxicity in the cells, treated with GO–PEG with NIR to prove the biocompatibility of the proposed nanocarrier. We further studied the prospective of GO–PEG in combination with NIR to modulate the activity of certain stress-responsive genes. Our results demonstrate the potential of GO–PEG bioactivity in the development of nanosystems for colorectal cancer treatments.

## 2. Materials and Methods

### 2.1. Preparation of Poly(Ethylene Glycol)-Modified Graphene Oxide (GO–PEG) and Physicochemical Characterization of NPs

Preparation of GO–PEG NPs was performed using pristine GO (Graphenea, Spain) and mPEG-NH_2_ (Abbexa Ltd., Cambridge, UK) following a previously established method of [38]. A comprehensive description of the PEGylation of GO and its physicochemical characterization was carried out in our previous publications [36,39]. Dynamic Light Scattering (DLS, Zetasizer, Malvern Instrument, Ltd., Worcestershire, UK) was used to determine particles size distributions, average particle size, zeta potential and polydispersity index (PDI) of GO and GO–PEG NPs; transmission electron microscope (TEM, JEM-2100, Tokyo, Japan) was used to analyze the nanoparticles’ morphology; and UV-Vis spectrophotometer (Specord 210 Plus, Edition 2010, Analytik Jena AG, Thuringia, Germany)—to measure adsorption spectra of both NPs in NIR region.

### 2.2. Cell Cultures, Media and Treatment Protocols

HT29 is a cell line derived from human colorectal cancer cells (ATCC, HTB-38) while Colon26 is derived from a mouse colon adenocarcinoma (ATCC, CRL-2638). Both cell lines were grown in Dulbecco’s modified Eagle’s medium (DMEM), supplemented with 10% (*v*/*v*) fetal bovine serum (Sigma-Aldrich, Darmstadt, Germany) and 1% (*v*/*v*) a mixture of antibiotics (104 IU penicillin and 104 μg streptomycin, Sigma-Aldrich, Germany). The cells were incubated at 37 °C with 5% CO_2_ and 95% humidity. Cells were passaged in the exponentially growing phase every second day, using 0.05% trypsin and 0.02% EDTA. Cells were seeded at a density of 2.5 × 10^4^ cells/well in 96- or 6-well plates and 24 h after seeding the cells were treated with 100 µg/mL GO or GO–PEG. Cells were grown under these conditions until 72 h during the timeline of the experiments.

### 2.3. Near-Infrared Irradiation

After an incubation period of 24 h with GO or GO–PEG NPs, the cells were irradiated for 15 min at room temperature using a NIR-based source (laser) with peak emission around 808 nm (NIR region) and irradiance of 1.5 W/cm^2^. For long-term assays (72 h), the cells were subsequently NIR-irradiated every 24th hour. Straight before the assays, the media containing GO and GO–PEG dispersions were removed, cells were washed with PBS, and all assays were performed as described in this section. To compare the effects of NIR irradiation, negative controls in the absence of the nanomaterials and without NIR irradiation were used.

### 2.4. Cell Proliferation Assays (WST-1)

Cells seeded in 96-well plates at a density of 2.5 × 10^4^ cells/well were incubated for 72 h. During cultivation the WST-1 assay was performed at three-time points: 24 h, 48 h and 72 h, to assess cell growth. Briefly, the medium with NPs was removed, the cells were washed with PBS and 100 μL medium with 10 μL WST-1 (tetrazolium salt 4-(3-(4-iodophenyl)-2-(4-nitrophenyl)-2 h-5-tetrazolium)-1,3-benzene disulfonate) were added to all wells. After 2 h incubation at dark, the optical density of the samples was measured at 450 nm using an ELISA reader Thermo Scientific Multiskan Spectrum (Thermo Scientific, Tokyo, Japan).

### 2.5. Fluorescence-Activated Cell Sorting (FACS) of Cells

#### 2.5.1. Cell Cycle Analyses after Staining with Propidium Iodide (PI)

Cell cycle analysis of Colon26 and HT29 cells, after 24 h and 72 h of cultivation was performed, as described previously [40]. Cells were fixed with 76% of cold ethanol and left at −20 °C for 24 h. After fixation cells were pelleted by centrifugation, washed in PBS buffer and treated with 100 µg/mL RNAse A for 30 min at 37 °C followed by staining with 50 µg/mL of PI for 30 min in the dark. A total of 50,000 cells were counted through flow cytometry, detecting red fluorescence at the excitation wavelength of 488 nm and the obtained data were analyzed by FlowJo™ software Version 10 Ashland (Becton, Dickinson and Company; 2019, San Diego, CA, USA).

#### 2.5.2. Mitochondrial Activity Analyses after Staining with Rhodamine 123 (Rh123)

FACS evaluation of the effect of GO, GO–PEG and NIR alone or the combination of NPs with NIR on the mitochondrial activity in Colon26 and HT29 cells was performed as described in [41]. To this aim, mitochondria were stained with Rhodamine 123 (Rh123), a lipophilic cationic fluorescent dye, which is routinely used to assess mitochondrial metabolism and activity [42,43,44,45] in individual cells by flow cytometry [36,41,46]. Depending on the respiration-driven membrane potential (ΔΨm), only mitochondria of viable cells incorporate the appropriate amount of Rh123. Briefly, adherent cells were dissociated by trypsinization, washed and resuspended in complete medium DMEM, pre-warmed at 37 °C. All samples were stained with 1 µg/mL of Rh123 for 30 min at 37 °C, collected by centrifugation at 5000 rpm for 5 min at 4 °C, washed twice with cold 1 × PBS, pH 7.0, placed on ice and immediately analyzed by Flow cytometry (BD FACSCalibur™ Instrument, Becton Dickinson). For a negative control group, before Rh123 staining, aliquots of cells were first incubated with 20 µM or 40 µM FCCP at 37 °C for 20 min. FCCP (carbonyl cyanide p-(trifluoromethoxy)phenylhydrazone) is a specific mitochondrial inhibitor that is a potent uncoupler of oxidative phosphorylation causing depolarization of mitochondrial membrane potential. To determine the dead cell population, 1 min before flow cytometry acquisition Rh123-stained cells were administrated with Propidium iodide (PI) to a final concentration of 2 µM. A total of 50,000 cells were acquired and the obtained data were analyzed by FlowJo™ as in the FACS experiments for assessing cell cycle progression. Forward (FSC) and side scatter (SSC) acquisition were performed in linear mode and used to detect and gate only viable cells [41]. Live cell populations were further analyzed for mitochondria-specific Rh123 incorporation by counting the FL1-H positive fluorescent cells while PI-stained dead cells were detected by FL3-H.

### 2.6. Genotoxicity Analysis by Single Cell Gel Electrophoresis (SCGE)

The method of Single Cell Gel Electrophoresis (SCGE) was used as previously described [46]. Colon26 and HT29 cells, after 24 h and 72 h of cultivation with GO or GO–PEG with and without NIR irradiation, were examined by neutral SCGE. The TriTek Comet Score Freeware v1.5 software (TriTek, Corp. Sumerduck, VA, USA) was used for SCGE results quantification. Three repetitions of the experiment were carried out and results are presented as MEAN ± STDV of the calculated Olive Moment parameter.

### 2.7. Fluorescent Microscopy Analysis of Mitochondria after Staining with Rh123

Several cationic, ∆Ψ-sensitive fluorescent dyes can be used for labeling mitochondria in living cells including Rhodamine 123 (Rh123) [45]. To investigate whether incubation of colorectal cancer cells with graphene nanoparticles with or without additional exposure to NIR caused any toxicity to mitochondrial function, cells were double-stained with 1 µg/mL Rh123 and 2 µM PI fluorophores for 30 min at 37 °C and 30 min at RT (room temperature), in the dark. Negative control cell groups were Colon26 cells treated with 20 µM FCCP and HT29 cells treated with 20 µM and 40 µM FCCP, for 20 min at 37 °C before being dual labeled. Imaging was performed under Leitz fluorescent inverted microscope Orthoplan, VARIO ORTHOMAT 2 (Vaughan, ON, Canada) using 450–490 nm bandpass filter and long-pass 515 suppression filter. Photo documentation was carried out with a built-in microscope Levenhuk^®^ M1400 Plus digital camera 14 Megapixels, Sensitivity, v/lux.sec @550 nm: 0.724 (Levenhuk, Inc., Tampa, FL, USA).

### 2.8. Gene Expression Analysis by RT-qPCR

Total RNA was isolated from the cultivated Colon26 and HT29 cells, treated with GO nanoparticles in combination with NIR irradiation for 24 h and 72 h, using Universal RNA Purification Kit (EURx), including the optional DNase I digestion step. This was followed by reverse transcription into cDNA of 280 ng DNase I-treated total RNA, using NG dART RT-PCR kit (EURx). Gene expression analysis was performed for the reference gene (GAPDH) and the genes of interest—ATM, TP53, BBC3 (PUMA), CDKN1A (p21), and RAD51. The used primers are described in Table 1. The reaction was carried out by the use of SG qPCR Master Mix (2×) (EURx), with 14 ng total RNA and 0.5 µM primer concentration, on Rotor-Gene 6000 (Corbett LifeScience). Three repetitions of the experiment were performed. The results were analyzed using the comparative CT method (ΔΔCT method) [47]. More than a 2-fold change in the expression level (up or down) compared to the calibrator (the respective untreated control group) was considered as significant.

### 2.9. Statistical Analysis

Data in this article were statistically analyzed by Microsoft Excel software version 10, (Microsoft, Redmond, WA, USA), in which bars represent the MEAN values of the calculated parameters ±STDV. Student’s *t*-test was performed, where the probability levels of 0.05 were considered statistically significant. Additionally, Dunnett’s test was conducted for proliferation activity assays of Colon26 and HT29 cells.

## 3. Results and Discussion

### 3.1. PEGylated Graphene Oxide Nanoparticles with Near-Infrared Laser Irradiation Proved Non-Toxic for Colorectal Carcinoma Cells

#### 3.1.1. Physicochemical and Biophysical Characteristics of GO and GO–PEG NPs

**Figure 1:**
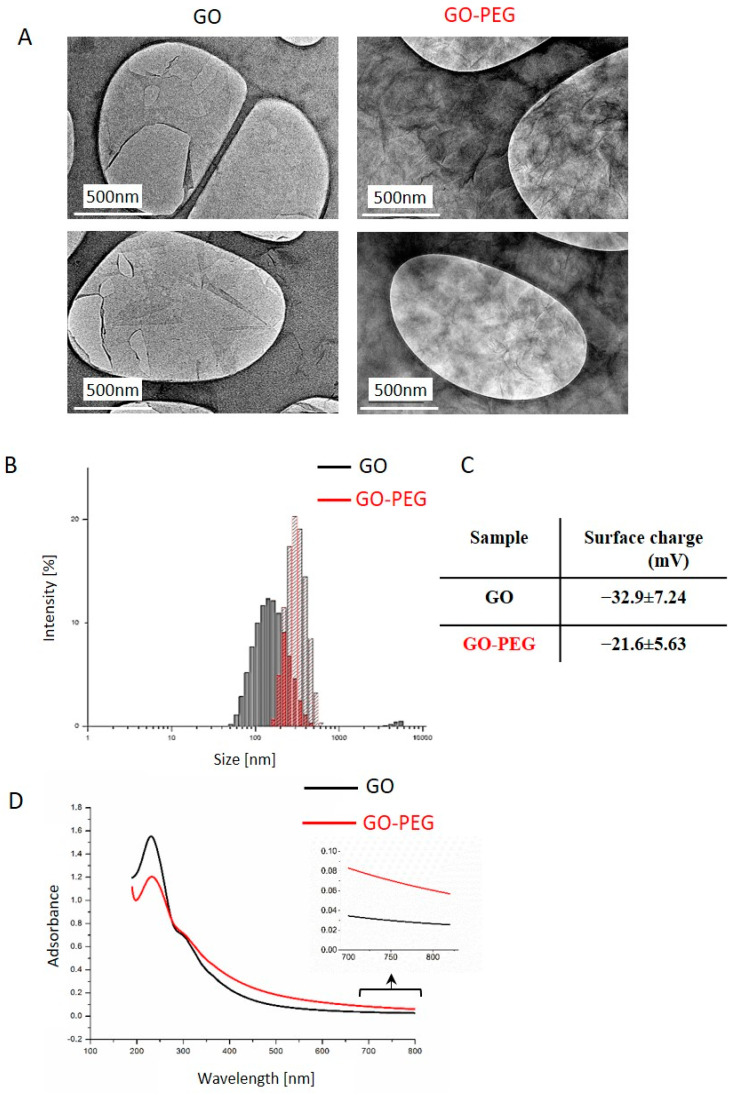
Physiochemical properties of GO and GO–PEG NPs. (**A**) Representative TEM images of GO and GO–PEG water dispersions, sonicated 1 h at 40 Hz (**B**) Size distribution analysis of GO and GO–PEG. Aqueous dispersions of GO and GO–PEG at 1 mg/mL were characterized by DLS after sonication using a particle size analyzer. The histogram shows the average values from triplicate measurements. (**C**) Zeta potential of GO and GO–PEG in water solution. (**D**) Characterization of GO and GO–PEG by ultraviolet-visible spectroscopy with an insert with the enlargement of the NIR region between 700–820 nm. At least three independent spectroscopic measurements were conducted for each sample.

This work aimed to evaluate the potential of GO–PEG nanoparticles to serve as a phototoxic switching nanocarrier system for colorectal cancer cells treatment. For this purpose, GO–PEG nanoparticles were synthesized by the method of [38] with some modifications. The detailed description of the preparation and detailed physicochemical characterization of both GO and GO–PEG NPs was already reported by us in [36,37]. In brief, we showed that the pristine GOs were negatively charged and appeared as thin and transparent sheets with relatively smooth surfaces (Figure 1A). The estimated average particle size of GO was 252.7 nm with a zeta potential of −32.9 mV (Figure 1B,C). In contrast, PEGylated GO nanoparticles were larger—324.6 nm, with a lower negative charge of −21.6 mV, and a wrinkled surface, which we accepted as a feature that favors their functionalization with anticancer drugs or other bioactive molecules. We consider that the detected differences in the size of both GO NPs could be due to the larger PEG moiety (0.35 kDa) and the replacement of the negatively charged -COOH group in GO molecules with neutral PEG molecules resulting in a lower negative ζ-potential. Both NPs showed a good absorbance in the NIR spectrum (at 808 nm) with a higher NIR absorbance of GO–PEG (Figure 1D, see the insert of the NIR enlargement section). In [37] we evaluated the outcomes of GO PEGylation on the structure and function of human blood components, especially on the morphology and the hemolytic potential of red blood cells (RBCs). We demonstrated a difference between the impact of pristine and PEGylated GO on blood components. Pristine GO had higher hemolytic activity and hematotoxicity, indicating that the PEGylation diminished the adverse effects of pristine nGO on human blood, probably by forming a shield around GO NPs.

Based on these physicochemical properties and the high optical absorbance especially of the modified GO, we consider both GO and PEGylated GO NPs as favorable candidates for phototoxic switching nanocarrier systems for colorectal cancer treatment. Therefore, we proceeded with detailed analyses of their bioactivity in colorectal carcinoma cells.

#### 3.1.2. PEGylated GO, Combined with NIR Irradiation, Are Non-Toxic for Colorectal Carcinoma Cells Regardless of the Cultivation Time

**Figure 2:**
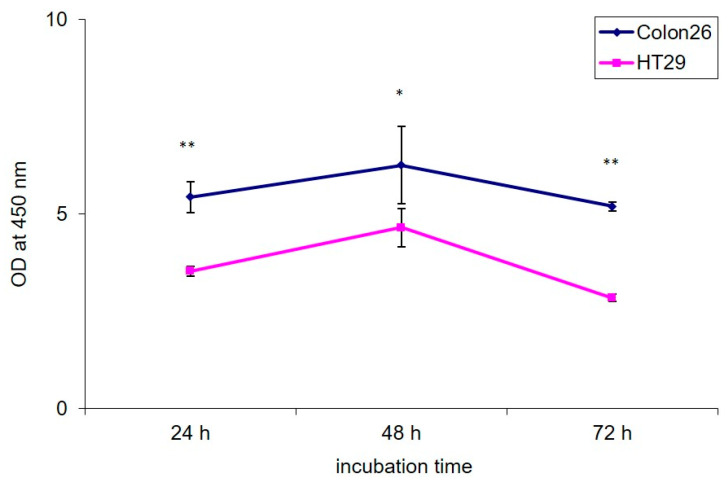
Proliferation activity of Colon26 and HT29 cells. Cells were seeded in 96-well plates at a density of 2.5 × 10^4^ cells/well and were incubated for 72 h. During cultivation the WST-1 assay was performed at three time points: 24 h, 48 h and 72 h, to assess cell growth. The optical density was measured at 450 nm using an ELISA reader. The error bars represent the standard errors of the mean OD at 450 nm assessed with WST-1 method (SEM). Statistically significant differences between the two cell lines in the OD were analyzed by Dunnett’s test and are denoted as * *p* < 0.01 and ** *p* < 0.0001.

For our study, two colorectal carcinoma cell lines were used: Colon26 and HT29, characterized by different proliferation potential and invasiveness [48]. These cell lines, though derived from mouse and human donors had a very well characterized malignant potential published by other authors [44]. Under their experimental design, the Colon26 cells appeared the most aggressive with 75.4% and 31.2% of cell migration and invasion compared to HT29 cells, which were less aggressive with 6.7% and 1.8% of migration and invasion calculated [44]. Therefore, in our further experiments, we chose these two types of colorectal cancer cells for comparison of the bioactivity of the studied nanomaterials. The used in this study concentration of 100 µg/mL was selected based on previously reported by us dose–response curves of Colon26 and HT29 cells incubated with different concentrations of GO and GO–PEG, where we did not observe any reduced viability in the range of 5–50 µg/mL [36]. All concentrations in the range of 100–500 µg/mL, however, inhibited cell viability. Therefore, for our experiments, we chose the lowest of these cytotoxic concentrations, i.e., 100 µg/mL. This concentration was also used in our previous study that preliminarily investigated the cytotoxic effect of GO and GO–PEG nanosheets under NIR laser irradiation in the same colorectal cancer cells. We found a strong synergic effect of GO–PEG and NIR on the migration of the low invasive HT29 cells [36]. For that reason, in the current research, we continued the detailed studies of the possible biological mechanisms of action of the studied nanomaterials by thoroughly examining their bioactivity. We measured the proliferation activity of the two cell lines for a period of 72 h. At three time points 24 h, 48 h and 72 h we checked the proliferation rates. The results are shown in Figure 2. Colon26 cells exhibited 1.5 up to 2 times higher proliferation rates compared to HT29 cells, kept until the last time point. On the 48th h-time point, we observed an increase in the proliferation of the two cell lines, which is normal regarding their malignancy. Regardless of this increase yet the differences in the way the two colorectal cancer cells proliferated remained unchanged in the favor of the high invasive Colon26. This result confirmed the dissimilar growth and metabolic potential of the two colon cancer cells in addition to their different migration and invasion potential observed from other authors too [48]. 

**Figure 3:**
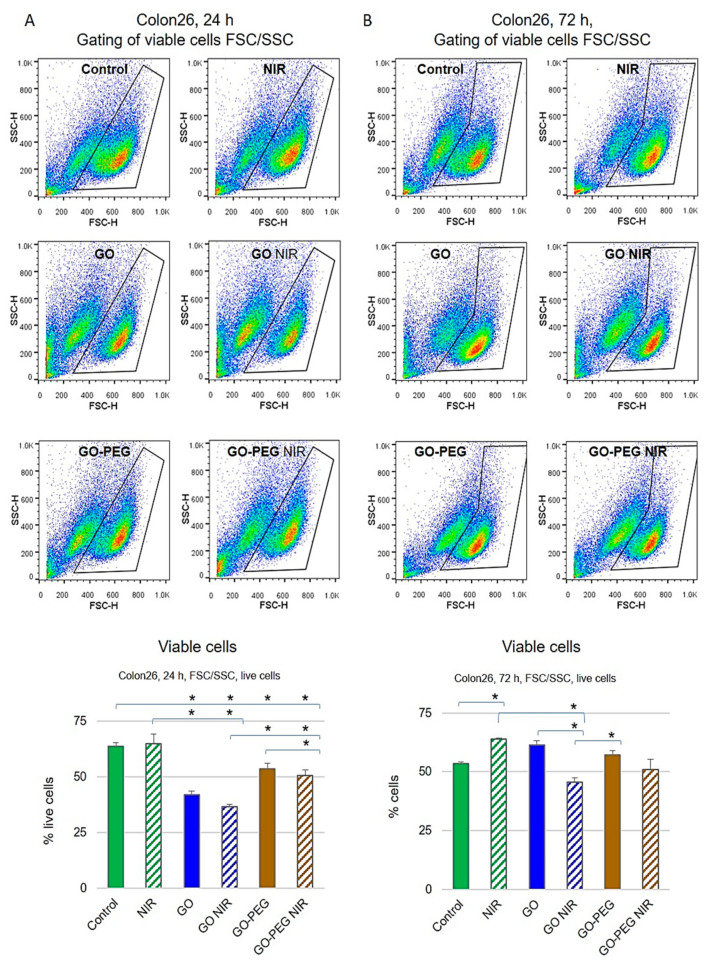
Cytotoxic effect of GO nanoparticles with or without NIR on Colon26 cells by FACS after staining of cells with Rh123. Representative dot-plots of FSC (forward side scattering) vs. SSC (side scattering) for Colon26 cells are presented. The population of viable cells was gated and the calculated percentage of live cells is given on the charts. (**A**) Colon26 viability after 24 h of cultivation with NPs. (**B**) Colon26 viability after 72 h of cultivation with NPs. Three repetitions of the experiment were carried out and results are the MEAN values of the calculated % of viable cells. Statistically significant differences are denoted with * *p* ˂ 0.05.

**Figure 4:**
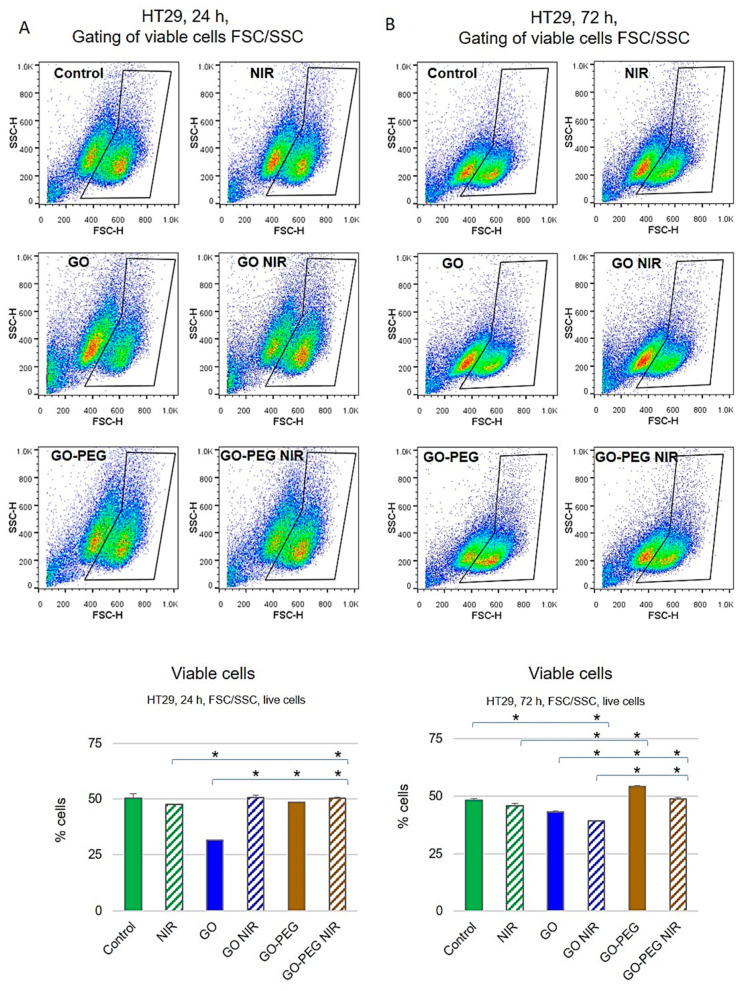
Cytotoxic effect of GO nanoparticles with or without NIR on HT29 cells studied by FACS after staining with Rh123. Representative dot-plots of FSC (forward scattering) vs. SSC (side scattering) for HT29 cells are presented. The population of viable cells was selected and the percentage of live cells is given on the charts as a function of all cells detected. (**A**) HT29 viability after 24 h of cultivation with NPs. (**B**) HT29 viability after 72 h of cultivation with NPs. Values are MEAN from three repetitions of the experiment and statistically significant differences are denoted with * *p* ˂ 0.05.

To further study the bioactivity of the studied nanoparticles in combination with NIR, we performed FACS analyzes with PI and Rhodamine 123 staining. Propidium iodide (PI) is a DNA binding fluorescent molecule that cannot enter passively into live cells with intact membranes. Conversely, the dead cells regardless of the mechanism of death become plasma membrane permeable for PI, which allows discriminating non-viable from viable cells by fluorescent microscopy or flow cytometry [49], while viable cells accumulate Rhodamine 123. FACS dot-plots for Colon26 and HT29 cells with the estimated percentages of viable cells according to cells FSC (forward scattering) and SSC (side scattering) distribution are presented in Figure 3 and Figure 4. In addition, Rh123 and PI fluorescence positive populations (data not shown), accounting for viable and nonviable cells, respectively, were detected. The results for the positive Rh123 Colon26 cells, i.e., viable cells are shown in Figure 3. Colon26 cells treated with GO alone or in combination with NIR radiation exhibited a 35% reduction of the percentage of viable cells after 24 h of cultivation compared to the non-treated control group (*p* < 0.05), while cells treated with GO–PEG and GO–PEG plus NIR had only 15% reduction in the percentage of viable cells, suggesting a recovery of the observed GO cytotoxicity when GO are PEG-modified and combined with NIR. The last was confirmed by the results with the samples, irradiated with NIR only, where cellular viability remained similar to that of non-treated cells (Figure 3A). After 72 h of cultivation, the viability of the NIR-treated Colon26 cells was unchanged, resembling the results from the 24 h of cultivation. An increase of near 20% of viable cells in comparison with the non-treated control group (*p* < 0.01) was also observed. In fact, except for NIR irradiated cells, which exerted the best survival rate, the observed alterations in the viability of all NPs-treated cells were statistically insignificant compared to the control group (*p* > 0.05) (Figure 3B). Interestingly, for the two studied time points, 24 h (Figure 3A) and 72 h (Figure 3B) the Colon26 cells treated with GO in combination with NIR exhibited the lowest percentage of viable cells. Importantly, PEGylated GO with and without NIR showed insignificant changes in the viable population of cells (Figure 3B).

Cell viability of HT29 cells after 24 and 72 h of cultivation was also measured via flow cytometry (Figure 4). As shown in Figure 4A, at 24 h of incubation, GO-treated HT29 cells had the lowest viability while under the other treatments no significant impact on the cell viability was observed (*p* > 0.05). Administration of HT29 cells with GO NPs for 72 h (Figure 4B) also resulted in a high percentage of dead cells, while the most toxic appeared the combination of GO with NIR irradiation (*p* < 0.05 vs. control untreated group). Treatment with GO–PEG and GO–PEG in combination with NIR had no effect on the vitality of HT29 cells for the two studied time points. The results for assessing the cellular viability of the two cell lines after treatment with the studied NPs with and without NIR by FACS demonstrated that both types of colorectal carcinoma cells were more sensitive to the treatment with GO alone or in combination with NIR regardless of the incubation period. This is in line with the findings of other researchers for the cytotoxicity of graphene oxide [50,51,52]. Conversely, the treatment of the cells with PEGylated GO with NIR led to insignificant cytotoxicity. These results are following other authors’ data that have demonstrated increased biocompatibility of PEGylated GO and thus its increased applicability in drug delivery [29,52]. Several studies reported strong evidence that functionalization of GO with poly(ethylene glycol) (GO–PEG) enhanced the solubility, dispersity, aqueous stability, biodistribution and cytocompatibility as well as antibacterial potency of the NPs [53]. This is one of the reasons why PEG-functionalized GO NPs were recognized to be more convenient for use in biomedical applications as carriers for hydrophobic anticancer drugs and NIR-coupled photothermal therapy [33,53]. However, some authors consider that the increased biocompatibility of PEGylated GO is overestimated slightly as some data point at strong immunological responses during therapy with GO–PEG [54]. Therefore, it is of utmost interest to trace the biological activities of PEGylated GO under our conditions and study in detail the molecular mechanisms, through which the PEG-modified graphene oxide therapy in combination with NIR could potentially affect cellular vitality. Hence, even though under our experimental conditions the PEGylated GO, combined with NIR irradiation, proved non-toxic for the two types of colorectal carcinoma cells regardless of the cultivation time we conducted experiments to further verify and elucidate these findings.

#### 3.1.3. Treatment with GO–PEG with and without NIR Slightly Impaired the Cell Cycle of Colorectal Cell Carcinoma Cells at 24 h of Incubation

**Figure 5:**
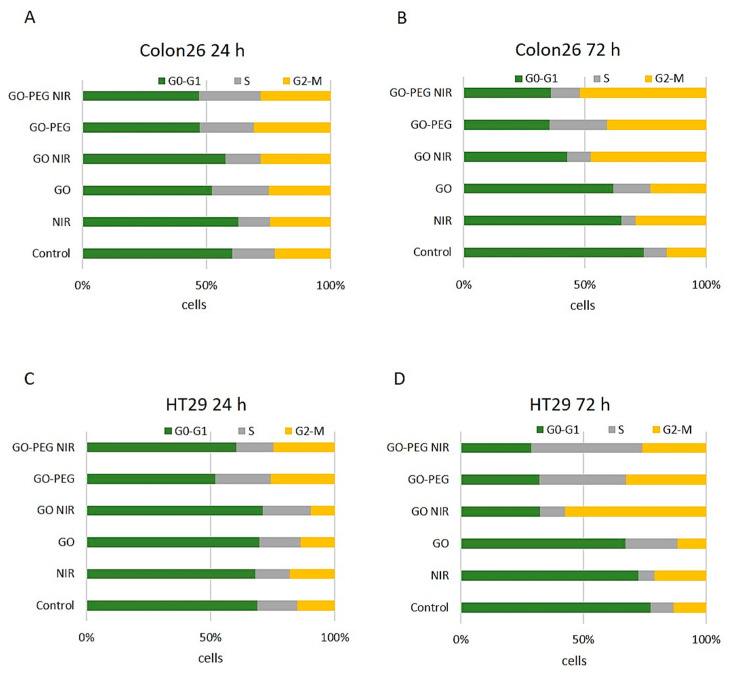
Cell cycle analysis of Colon26 and HT29 cells after PI staining and treatment with GO and GO–PEG NPs with and without NIR via FACS. (**A**) Distribution of Colon26 cells cultivated for 24 h in the presence of NPs with and without NIR irradiation in the cell cycle phases. (**B**) Distribution of Colon26 cells cultivated for 72 h in the cell cycle phases. (**C**) Distribution of HT29 cells cultivated for 24 h in the presence of NPs with and without NIR irradiation in the cell cycle phases. (**D**) Distribution of HT29 cells cultivated for 72 h in the cell cycle phases. Values are MEAN of % of cells from three repetitions.

Colon26 and HT29 cells were further studied by FACS for assessment of cellular progression through the cell cycle phases after staining with Propidium Iodide (PI). Results from the FACS analyzes given as the percentage of cells in the cell cycle phases are shown in Figure 5. Figure 5A shows Colon26 cells after 24 h of cultivation. The results demonstrated a slight decrease in the G0-G1 cell population and a reciprocal increase in the S-phase cell population after treatment with GO, GO–PEG and GO–PEG NIR. Exposure to NIR alone or in combination with GO did not affect the cell cycle progression. A minor increase in the percentage of cells in G2-M was observed in Colon26 cell culture exposed to GO–PEG and GO–PEG NIR at 24 h (Figure 5A). HT29 cells treated for 24 h (Figure 5C) showed the same tendency with a slight decrease in cells in the G0-G1 cell cycle phase under treatment with GO–PEG with and without NIR. An increase in the G2-M cell cycle phase was detected under the same treatments. The trend was similar with Colon26 cells at this time point.

As expected, the more pronounced influences of the applied treatments on the cell cycle progression were detected at 72 h for both Colon26 and HT29 cell lines (Figure 5B,D). For Colon26, the G0-G1 cell population decreased across all experimental groups while the percentage of cells in the G2-M cell cycle phase increased, suggesting a slight G2-M cell cycle arrest. These changes were most explicit in GO–NIR, GO–PEG and GO–PEG NIR treated groups (Figure 5B). Irradiation of HT29 cells with NIR did not influence significantly the cell cycle at 72 h in comparison to the non-treated control cells (Figure 5D). The treatment with NPs led to the accumulation of HT29 cells in a different phase of the cell cycle depending on the NP type and NIR exposure. Administration of GO resulted in the accumulation of HT29 cells in the S phase while the combination of GO with NIR caused an increase in the percentage of cells in the G2-M population. HT29 cultures exposed to GO–PEG alone or in combination with NIR demonstrated an accumulation of cells predominantly in S and to a less extend in G2-M phases. Shortly, the two cell lines, Colon26 and HT29 showed a reduction in the percentage of cells in G0-G1 on the 72nd—time point and an increase in cell populations in S and/or G2-M cell cycle phases (Figure 5B,D). The last is an indication for a slight cytostatic effect of GO–NIR, GO–PEG and GO–PEG in combination with NIR on HT29 cells at this time of cultivation. 

These results are consistent with other authors’ results that demonstrate the biocompatibility of different nanomaterials [17,55]. The authors repot novel and smart nanosystems and proved their good specificity and stability, visual detection of drug loading, responsive biodegradation and drug release, effective cancer chemotherapy and anti-migration.

### 3.2. Insignificant Genotoxicity of GO–PEG NPs in Combination with NIR for Colon26 and HT29 Cells after 24 h of Cultivation

**Figure 6:**
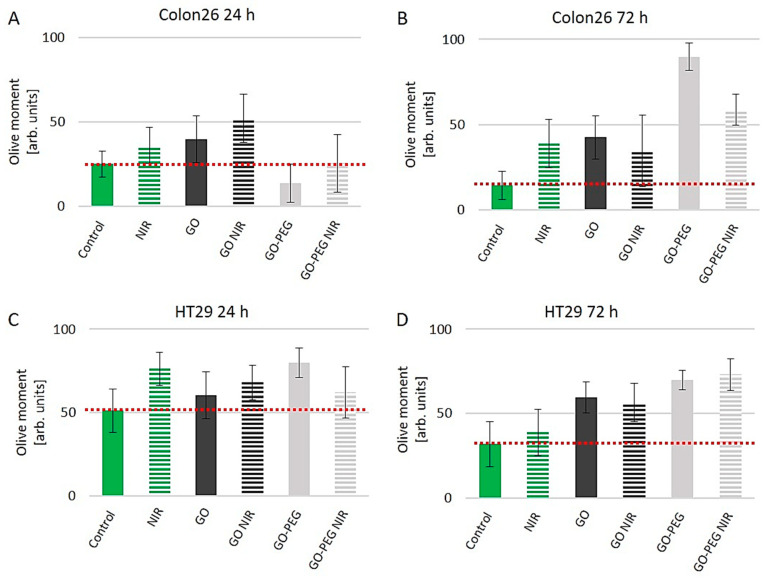
Investigation of the genotoxic potential of GO and GO–PEG with and without NIR irradiation on Colon26 and HT29 cells by the method of SCGE. (**A**) The parameter Olive Moment calculated for Colon26 cells cultivated for 24 h in the presence of the NPs with and without NIR irradiation. (**B**) The parameter Olive Moment calculated for Colon26 cells cultivated for 72 h in the presence of the NPs with and without NIR irradiation. (**C**) The parameter Olive Moment calculated for HT29 cells cultivated for 24 h in the presence of the NPs with and without NIR irradiation. (**D**) The parameter Olive Moment calculated for HT29 cells cultivated for 72 h in the presence of the NPs with and without NIR irradiation. The dotted red lines denote the threshold, above which we detect genotoxicity. Values of the Olive moment are the MEAN±. STDV from three repetitions of the experiment.

It is accepted that for all bioengineered nanomaterials the biocompatibility is of high importance. Furthermore, the interactions of the nanomaterials with central biomolecules such as DNA, RNA and proteins are also crucial. Consequently, to further study the bioactivity of GO–PEG in combination with NIR we performed experiments aiming to address the interactions of these NPs with DNA. Exposure of cells to different genotoxic stress such as radiation, drugs or nanoparticles leads to DNA damage and triggers subsequent cascades of DNA repair signaling pathways to control cancer cell cycle arrest and cell fate [56]. To check whether the as-developed therapy of GO–PEG and NIR induce DNA damage in the studied colorectal carcinoma cells we performed the Single-Cell Gel Electrophoresis assay (SCGE), also named Comet Assay, which is a widely used method for detection of DNA damage at a single-cell level. The method is vastly applicable in various genotoxicity studies of nanomaterials [57]. Briefly, the method detects extended DNA loops toward the anode during the electrophoresis step, which are a result of induced DNA damage. The neutral version of the method as in our experiments detects mainly double-stranded DNA breaks, which are generally referred to as DNA damage due to apoptosis [58]. Comet Assay data quantitation was performed with the software CometScore [59]. The Comet Assay parameter “Olive moment” was used as a measure of DNA damage, respectively genotoxicity. Data quantitation of the parameter “Olive Moment” for Colon26 cells and HT29 cells exposed to 100 µg/mL of GO and GO–PEG NPs with and without NIR for 24 and 72 h of cultivation are presented in Figure 6. A red dotted line represents the threshold, over which we consider the presence of genotoxic effect as a result of GO treatments.

Colon26 cells after 24 h of cultivation under the treatment protocols in this study appeared more sensitive to the genotoxic action of NIR alone, GO and GO in combination with NIR as seen in Figure 6A. The detected change in the Olive Moment values in comparison to the control for NIR, GO and GO–NIR demonstrated a 1.4, 1.6 and 2-fold increase, respectively. The observed genotoxicity in this row of treatment was the highest at cells handled with GO in combination with NIR irradiation and seemed to be a result of the cumulative genotoxic effect of all treatments. Importantly, the exposure of these cells to GO–PEG ranged from lack of genotoxicity to a very faint genotoxicity level when GO–PEG was combined with NIR (Figure 6A). We further found a similar influence of genotoxicity for NIR, GO and GO in combination with NIR on Colon26 DNA after 72 h of cultivation, detecting respectively 2.7, 3.0 and 2.4-fold higher “Olive Moment” values than the controls (Figure 6B). However, the exposure for 72 h of Colon26 to GO–PEG alone induced a 6-fold increase in the detected genotoxicity and a 4-fold increase in genotoxicity, when cells were treated with GO–PEG NIR in comparison to the nontreated group. The obtained results revealed DNA damage in Colon26 cells exposed for 72 h to GO–PEG NPs alone or in combination with NIR irradiation in comparison to the cells treated for 24 h only. The increased DNA damage caused by GO–PEG NIR correlated with the altered distribution of cells throughout the cell cycle phases, with a decrease in G0-G1 population and elevation of G2-M population suggesting a G2-M arrest (Figure 5B). Therefore, the putative mechanism of action of GO–PEG with or without NIR after long-term application, including the prolonged cultivation and longer irradiation time, implied enlarged DNA damage.

When the genotoxic effect of the same treatment procedures on HT29 cells was analyzed we observed that these cells also proved sensitive to the DNA damaging action of GO, GO–PEG with and without NIR irradiation, regardless of the cultivation and treatment time (Figure 6C,D) as opposed to the finding for Colon26. These results confirmed our preliminary results with the biological activity of GO–PEG as reported in [36], where we detected specific cytotoxic and cell proliferation inhibiting effects of GO–PEG with and without NIR on these particular types of colorectal cancer cells. If we compare the two samples cultured for 24 h and 72 h, in which we apply NIR irradiation alone, it could be assumed that HT29 cells were more susceptible to DNA damage by NIR irradiation at the first time point (Figure 6C). With the cultivation and irradiation time increasing to 72 h this NIR-DNA damage weakness decreased with two folds (Figure 6D; 52% increase for 24 h and 22% increase for 72 h vs. appropriate control group). HT29 cells demonstrated higher overall DNA damage than the Colon26 cells, moreover, HT29 showed greater sensitivity to GO–PEG NPs as were the results from our preliminary results studying the bioactivity of these NPs [36]. However, at the 24 h of cultivation, the NIR irradiation decreased the DNA damage in GO–PEG treated HT29 cells by 1.2-fold (Figure 6C), while a longer NPs treatment (for 72 h) increased the photosensitivity of HT29 cells resulting in higher DNA damage in NIR-treated H29 cells (Figure 6D). The detected genotoxicity of GO–PEG with and without NIR was increased by 2.3 folds in comparison to the control cells and reflected the accumulation of a vast proportion of cells in the S and G2-M phases of the cell cycle (compare with Figure 5D).

Previous studies have also shown that exposure to graphene oxide and rGONR–PEG caused concentration and size-dependent DNA damage in different cancer cells including human ovarian cancer cells, human *Glioblastoma multiforme* cells (GBMU87), human alveolar adenocarcinoma cells (A549), CaCO2 and Vero cell lines [51,60,61,62,63], suggesting that GO and GO–PEG have genotoxic effects on cells, depending on their nature and treatment protocols. These results signified that the cyto- and genotoxicity of graphene materials should be carefully studied before combining with the other therapeutic approaches such as photothermal therapy [64]. Our studies demonstrated that PEGylation of GO alone and in combination with NIR had none to little DNA damaging activity in Colon26 and HT29 cells, respectively, after 24 h of cultivation and higher genotoxicity after 72 h of cultivation. In addition, it appeared that in a short-time treatment the low proliferating (Figure 2) and low invasive HT29 cells were more susceptible to the genotoxic action of GO–PEG ± NIR than the high invasive Colon26 cell line (Figure 6A,C). When the treatment and cultivation continued for a longer period (72 h), the inverse relationship was observed Colon26 cells showed a higher rate of DNA damage (6.4 and 4.2-fold increase for Colon26 vs. 2.2 and 2.3-fold increase for HT29 cells after treatment with GO–PEG and GO–PEG NIR, respectively; Figure 6B,D). This is a very important finding as it allows discrimination of the mode of action of GO–PEG on high and low invasive colorectal carcinoma cells especially in the light of its genotoxicity. Moreover, these data tolerate hypotheses for the use of this nanocarrier in these two types of cancer cells in different manners: one to assure cytotoxicity and death in the cancer cells and the other to permit the development of a smart nanocarrier system, in which the nanocarrier itself does not induce damage to the targeted cells, nor to the surrounding ones. This hypothesis, though, requires future research and is an intriguing field for future explorations.

### 3.3. PEGylated Graphene Oxide Nanoparticles Combined with Near-Infrared Laser Irradiation Has Little Mitotoxicity in Colorectal Carcinoma Cells

**Figure 7:**
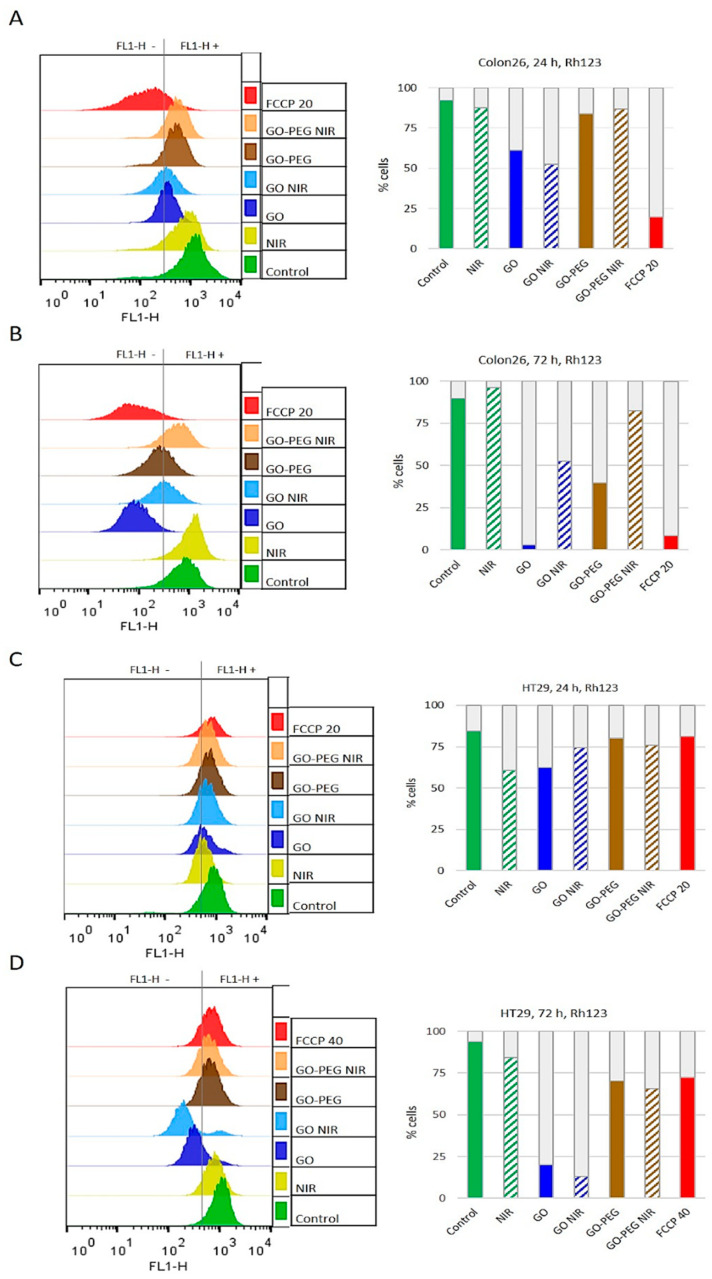
Mitotoxicity of GO nanoparticles and NIR by Rhodamin123 staining assessed by FACS observation. The mitochondrial activity was detected by FACS based on the incorporation of ∆Ψ-sensitive Rh123 fluorescent dye in viable cells. Histograms represent Rh123 fluorescence acquired by flow cytometer using FL1-H detector. The charts show the distribution of gated viable cells (FSC/SSC) according to the intensity of their Rh123 (FL1-H) fluorescence. (**A**) Colon26 cells after 24 h incubation with NPs. (**B**) Colon26 cells after 72 h of NPs treatment. (**C**) HT29 cell—24 h after incubation with NPs. (**D**) HT29 cells—72 h of treatment with NPs.

To evaluate Colon26 and HT29 cellular responses to GO and GO–PEG with and without NIR irradiation we continued our studies with analyses of mitochondria in the studied colorectal carcinoma cell lines. Cells with and without treatment with the discussed here nanomaterial were stained with Rhodamine 123 (Rh123). Next, cells were analyzed by FACS. Rh123 is a fluorescent dye that specifically incorporates within mitochondria due to the transmembrane potential of these organelles in living cells [65]. The results of these studies are shown in Figure 7. Histograms from the flow cytometry assays demonstrating the Rh123 fluorescence of Colon26 and data quantitation are shown in Figure 7A for 24 h of cultivation and in Figure 7B for 72 h of cultivation. For HT29 these histograms together with data quantitation are displayed in Figure 7C for 24 h and Figure 7D for 72 h. To abolish the mitochondrial membrane potential (MMP), before Rh123 staining cell aliquots were pre-treated with FCCP and these samples were used as a negative control group, i.e., cells with disrupted mitochondrial function. As expected, FCCP dramatically abrogated Rh123 uptake, indicative of abolished MMP and consequently impaired mitochondrial respiratory function.

In Colon26 cells at both time points, FCCP led to decreased accumulation of Rh123 by the cells with 79% at the 24 h-time point (see the chart with data quantitation in Figure 7A), and with 90.5% at 72 h-time point vs. non-treated control cells (Figure 7A,B, red vs. green). Amongst the studied effect of GO NPs on Colon26 mitochondrial activity, the most potent effect with 42% inhibition of the mitochondrial activity at 24 h was detected for GO in combination with NIR (Figure 7A, blue upward diagonals on the chart). GO–PEG treatment alone or combined with NIR irradiation led to a mitochondrial fitness comparable with that of the NP-less control group for the 24 h of cultivation (Figure 7A, see brown vs. green columns). As for Colon26 cells cultured for 72 h, the most significant drop, reduction with 96%, in the Rh123 positive population, was detected after treatment with GO NPs alone (Figure 7B, blue), while only 2.8% of the alive cells still retained an intact MMP. However, functionalization of GO NPs with PEG was much less mitotoxic giving 40% Rh123 fluorescent cells and NIR irradiation further increase the proportion of cells with uncrippled/unaffected MPP to 82%. (Figure 7B). PEGylated GO NPs especially in combination with NIR were observed to be much more mitocompatible for Colon26 cells than unmodified GO irrespective of the cultivation period, 24 h or 72 h.

Unexpectedly, the fluorescence of FCCP-treated HT29 cells at 24 h was unaffected exhibiting Rh123 uptake similar to that of untreated control cells (Figure 7C, green vs. red). A reduction of only 22% in HT29 Rh123-positive cells at 72 h was detected following the addition of 40 µM of the MMP inhibitor FCCP as seen in Figure 7D. This is an interesting observation, demonstrating a different mitochondrial sensitivity and functioning in these types of colorectal cancer cells. The highest reduction in the MMP in the population of HT29 cells at 24 h of cultivation, (though considered minor) a reduction was observed after application of NIR with 28% and GO only with 26% (Figure 7C, green upward diagonals and blue bars).

The most detrimental to the mitochondrial activity in HT29 cells cultured for 72 h were the treatments with GO only and GO in combination with NIR, showing a reduction in the stained mitochondria with 86% and 78%, respectively (Figure 7C,D, blue and blue upward diagonals). Notably, HT29 cells treated with GO–PEG and GO–PEG combined with NIR irradiation showed little mitotoxicity effect on the two-time points (Figure 7C,D, brown and brown upward diagonals columns). The detected reduction in the Rh123 uptake of the cells was around 10% only for 24 h, and around 30% for 72 h relative to the control non-treated group.

The comparison of Rh123 uptake in Colon26 and HT29 cell lines at 24 h and 72 h showed that NIR had a clear beneficial effect only for highly proliferating Colon26 cancer cells cultivated for 72 h in both applications alone or combination with NPs when compared to the respective non-irradiated group. NIR irradiation itself did not affect significantly the mitochondrial activity of 24 h Colon26 propagated cells nor that of HT29 cells for the whole period. It is also worthy to note that, the two cell lines were much more sensitive to GO and GO–NIR experiencing severe mitochondrial toxicity. Summarizing the obtained results allowed us to build a power-order of treatments according to their mitotoxicity (from lowest to the highest percentage of Rh123-fluorescent, i.e., viable cells):
For Colon26 at 24 h:GO NIR > GO > GO–PEG > GO–PEG NIR > NIRFor Colon26 at 72 h:GO > GO–PEG > GO NIR > GO–PEG NIR > NIRFor HT29 at 24 h:NIR > GO > GO NIR > GO–PEG NIR > GO–PEGFor HT29 at 72 h:GO NIR > GO > GO–PEG NIR > GO–PEG > NIR

This order for the mitotoxicity of the applied treatments reflects the cyto- and mitoxicity of the applied treatments on colorectal carcinoma cells. The different treatments have peculiar outcomes affecting particular cellular structures and functions of colorectal carcinoma cells. We could resume that the graphene derivative GO–PEG alone or combined with NIR irradiation demonstrated no apparent cyto- and mitotoxicity, thus suggesting higher biocompatibility and potential as a drug carrier.

**Figure 8:**
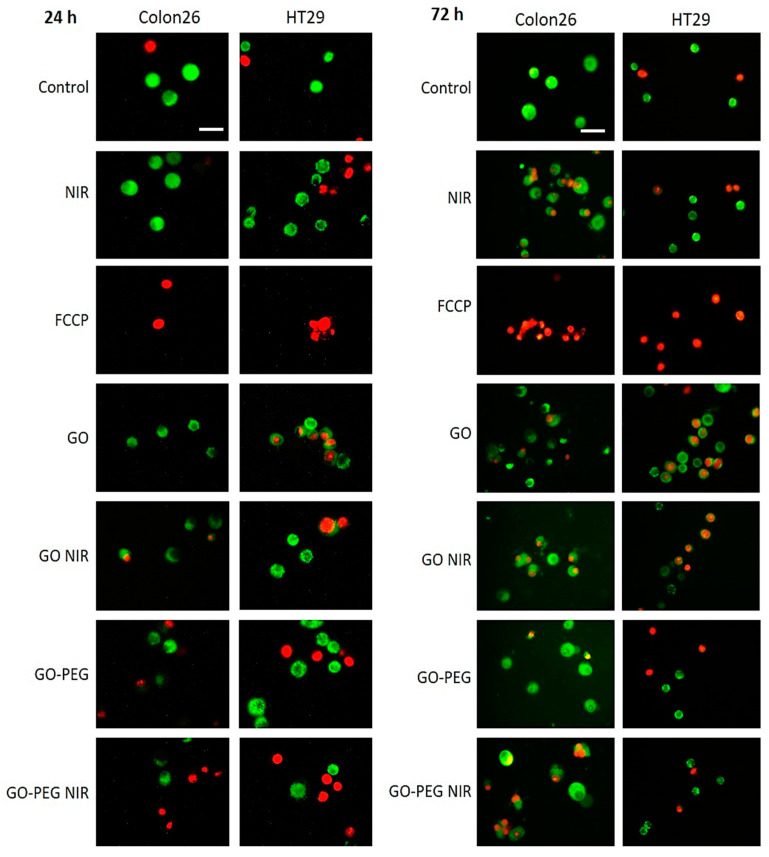
Fluorescent microscopy of mitochondrial morphology in Colon26 and HT29 cells incubated with GO and GO–PEG with and without NIR irradiation. Cells were stained with Rh123 at the two tested time points, 24 h and 72 h, and were visualized under an epifluorescent microscope. Representative epifluorescence images after staining with Rh123 and PI are displayed. Viable cells are green, whereas dead cells are red, due to accumulation of Rh123 and PI, respectively. White bars stand for 100 µm.

The cells analyzed by FACS after Rh123 and PI staining were observed under an epi-fluorescent microscope. Representative images are shown in Figure 8. The green cells denote viably and with active mitochondria cells, while the red is dead. What catches the attention is the FCCP inhibitor, whose application led to a drastic lethality among cells, as well as to an abrogated mitochondrial activity. NIR irradiation potentiated MMP in Colon26 cells at 24 h, with a little reduction in the percentage of green cells at 72 h. In both cases, NIR irradiation potentiated the mitochondrial activity in the studies cells. For HT29 cells NIR was harsher at 24 h while at 72 h led to a recovery of MMP and an increase in the number of green cells. Under all conditions and in the two studied cell lines GO–PEG alone had a moderate to little mito- and cytotoxicity, and the mitochondrial activity remained or was recovered close to the level of the control untreated group when GO–PEG was combined with NIR irradiation.

### 3.4. PEGylated Graphene Oxide Nanoparticles with Near-Infrared Laser Irradiation Modulate the Activity of Stress-Responsive Genes in Colorectal Carcinoma Cells

DNA damage activates numerous genes triggering multiple DNA repair pathways. Double-strand DNA breaks (DSBs) are repaired by two distinct pathways, non-homologous end joining (NHEJ) and homologous recombination (HR) pathways. To study the effect of GO–PEG with and without NIR on the activity of key stress-responsive genes we studied the expression of genes, involved in ATM-dependent homologous recombination DNA repair pathway. DSBs signals activate the Ataxia–telangiectasia-mutated (ATM) kinase, which in turn phosphorylates numerous effector proteins for DNA damage response such as RAD51, BRCA1 and TP53 genes products [66]. Further, p53 protein activates cyclin-dependent kinase—CDKN1A (p21) and Bcl-2-binding component 3 (BCC3) (PUMA) genes, resulting in G1 cell cycle arrest and mitochondrial dysfunction, respectively, both leading to apoptosis. Therefore, the first gene activity that we studied was the ATM gene, which encodes the ATM protein—a DNA damage response (DDR) signal transducer, recruited by the DNA damage sensors the core histone protein variant H2AX and the MRN complex (Mre11-Rad50-Nbs1) to extend DNA damage signalling.

**Figure 9:**
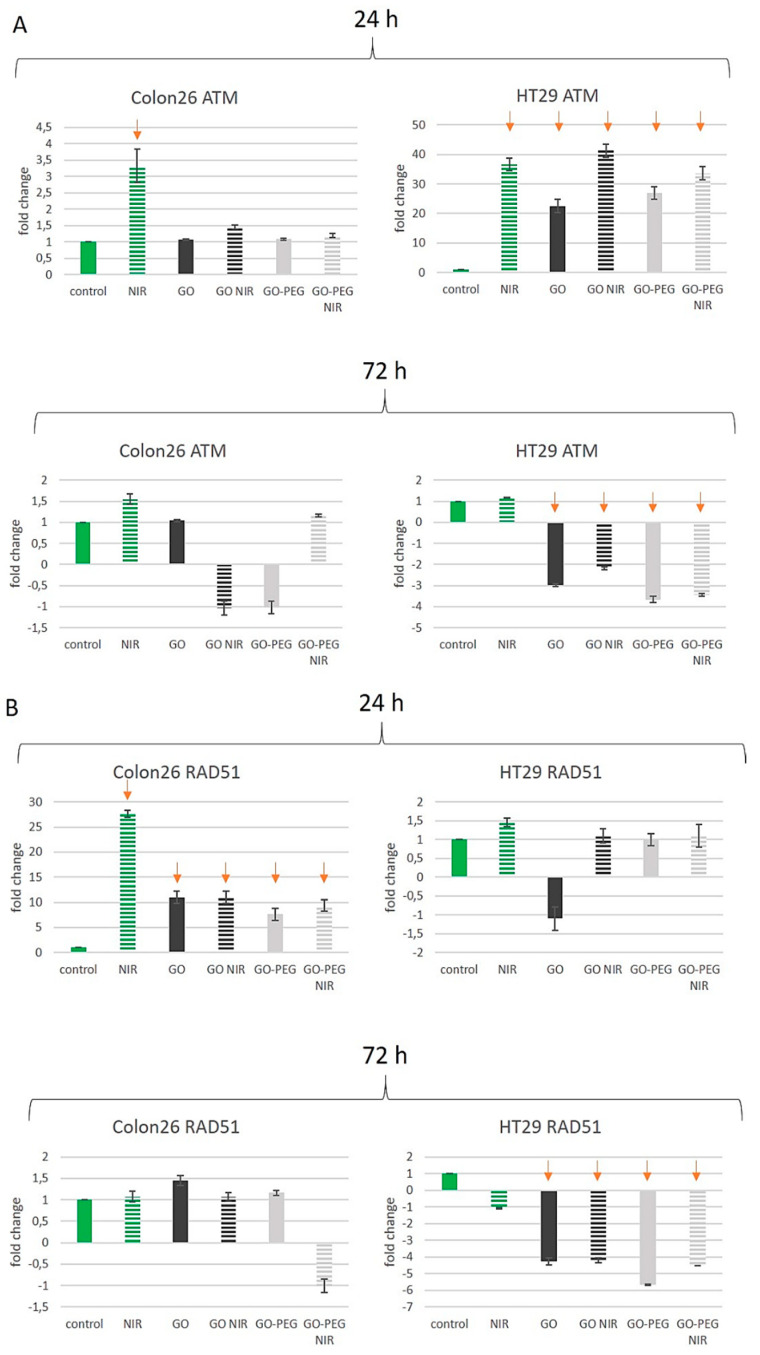

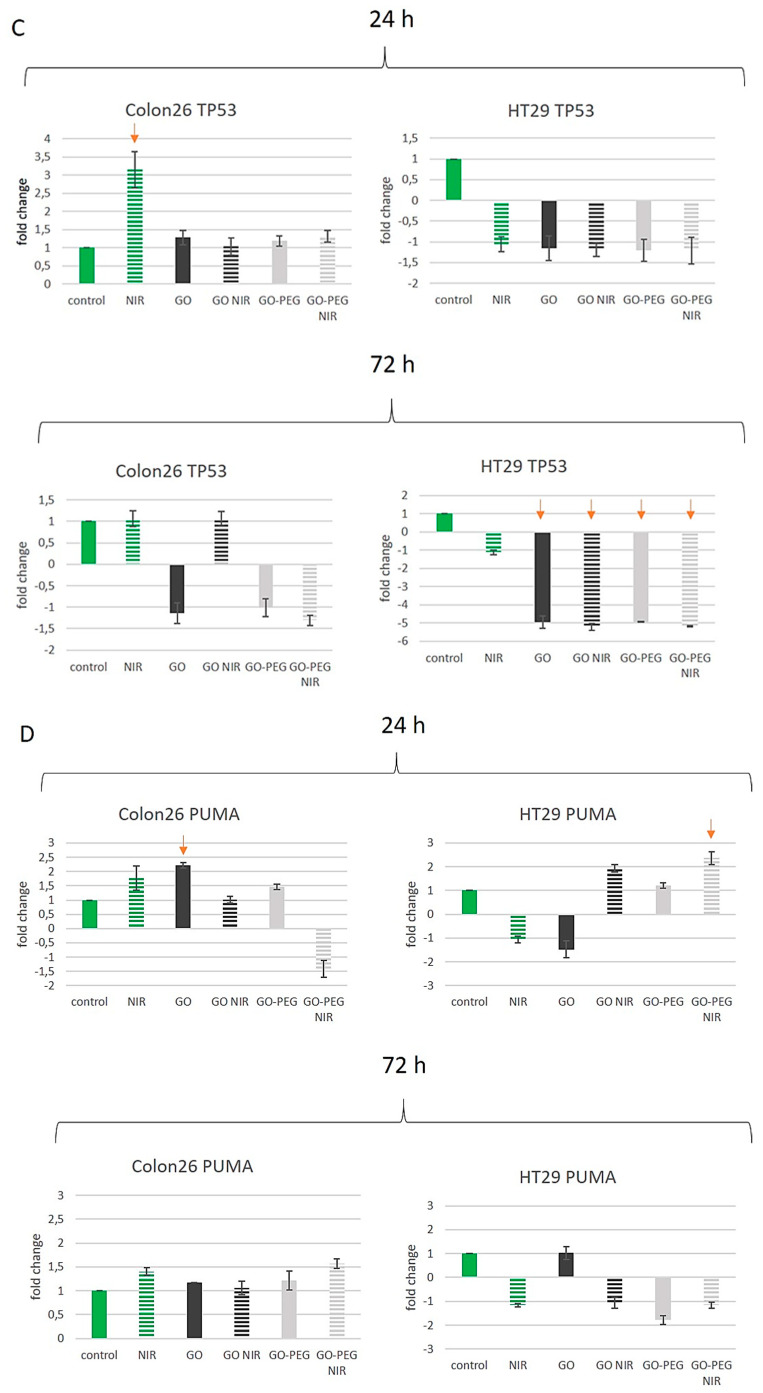

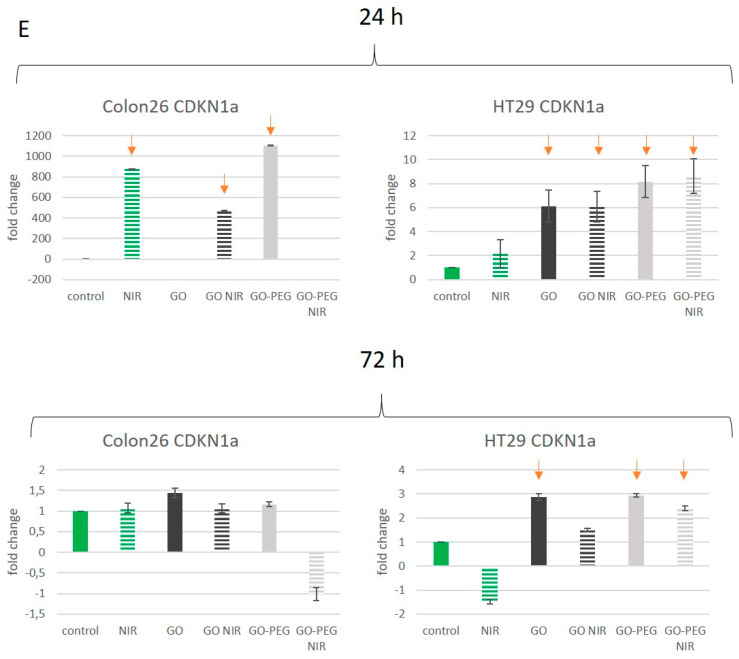
RT-qPCR for studying the effect of PEGylated GO nanoparticles with near-infrared laser irradiation on the activity of stress-responsive genes in colorectal carcinoma cells. (**A**) Fold change in the expression of the ATM gene. (**B**) Fold change in the expression of RAD51 (**C**) Fold change in the expression of TP53 (**D**) Fold change in the expression of BCC3 (PUMA) (**E**) Fold change in the expression of CDKN1A (p21) Three repetitions of the experiment were conducted and values represent the fold change in the expression of the studied genes as MEAN ± STDV. Red arrows denote significant alterations in the activity of certain genes after GO NPs treatment with and without NIR irradiation.

The ATM gene is frequently mutated in lymphoid malignancies, colorectal cancer (CRC), as well as in a variety of other solid tumours. ATM mRNA expression level in colorectal cancer tissues was higher compared to the one in normal mucosa tissues and adjacent non-cancerous tissue and was linked to apoptosis regulation and damaged cell repair [67]. ATM mRNA expression and the degree of differentiation of colorectal cancer were found to be negatively correlated, while there was no found connection with the expression levels and age, sex, tumour invasiveness, metastasis in the lymph nodes or clinical stage [68]. In our study, we found cell- and time-dependent differences in ATM expression levels after GO NPs treatments with and without NIR exposure. In Colon26 cells, the 24 h-exposure to GO and GO–PEG with and without NIR did not influence the expression level of ATM (Figure 9A, Colon24, 24 h). NIR irradiation, however, induced higher expression of ATM with three folds at 24 h and 1.5 folds at 72 h in Colon26 cells. Exposure to GO, GO–NIR, GO–PEG or GO–PEG NIR did not change ATM mRNA level at 24 h and at 72 h (Figure 9A, ATM, Colon26). The only treatment that caused more than a 2-fold change of ATM transcript compared to the respective calibrator sample (control for each tested period) was NIR irradiation of Colon26 at 24 h. For all other treatments, the change was less than 2-fold and therefore considered insignificant. In HT29 cells the effect of NIR irradiation alone led to a 35-fold increase in ATM expression levels at 24 h of cultivation. At 72 h the expression of ATM in these cells remained unchanged (Figure 9A, HT29 cells). The treatment with GO NPs only led to an increase in ATM expression at 24 h with more than 20 times in respect to the control untreated group. Oppositely, in 72 h-treated samples with GO suppressed the expression of ATP was suppressed with three folds, suggesting that these cells at 72 h were very sensitive to the genotoxic action of this type of treatment as seen in the results reported in Figure 6. PEGylation of GO and its combination with NIR led to the same increase in ATM mRNA expression levels as in NIR-irradiated cells only at 24 h. At 72 h of cultivation and treatment of HT29 cells with GO–PEG with and without NIR the tendency was the opposite. A reduction of 3 to 4-fold in the ATM mRNA expression levels was observed (Figure 9A, HT29, 72 h). A noticeable difference in the expression of ATM was detected, for Colon26 cells only irradiation with NIR at 24 h caused any considerable upregulation of the gene (3-fold); for 24 h cultured HT29 cells each one treatment increased more than 20-fold mRNA transcript while for 72 h cultured HT29 cells the ATM transcription was down-regulated upon all treatments except for NIR.

Our results with ATM-mRNA expression levels pointed to a negative correlation in the effect of the applied treatments on the regulation of ATM mRNA expression level and the tumour invasive potential of colon cancer cells. At 24 h of cultivation, only HT29 cells exhibited strong upregulation (more than 20-fold) of ATM transcription across all experimental groups, indicating that these cells experience higher genotoxic stress during different treatment protocols and thus maintain high levels of ATM expression. These results are confirmed by other authors too that also show [68] a negative correlation between the expression of ATM mRNA and tumour invasion [68].

The formation of Rad51 nucleofilaments is the main characteristic of homology-directed repair (HDR) of DNA [69]. RAD51 is a eukaryotic gene, encoding an enzyme member of the Rad51 protein family. It plays a key role in DNA repair through homologous recombination (HR). Rad51 is recruited to sites of DNA damage directly by BRCT-2 through interaction with conserved BRCT motifs to stabilize the Rad51 nucleoprotein filament on the ssDNA end of DSBs [70]. It was reported that BRCA1 regulates the Rad51 recruitment, the BRCA1-BARD1 dimer enhances the RAD51 recombinase activity and promotes RAD51-mediated pairing of homologous sequences [71,72]. To repair a damaged region on the DNA sequence, Rad51 facilitates strand transfer between the two homologous sequences [73]. Together with RAD51 and XRCC3, under oxidative stress conditions, it also regulates the mitochondrial DNA copy number [74]. Numerous studies report that RAD51 is over-expressed in different cancers. In many of these studies, elevated expression of RAD51 correlated with decreased patient survival [75,76].

There are data demonstrating the down-regulation of RAD51 expression in cancers [77]. What we found in our study was a 27-fold overexpression of RAD51 in NIR-irradiated Colon26 cells at 24 h and an increase of about tenfold for RAD51 mRNA expression in GO and GO–PEG treated cells, with and without the application of NIR (Figure 9B, Colon26, 24 h). However, after 72 h incubation of Colon26 cells with the tested NPs the RAD51 transcript levels decreased to the level of untreated cells in all applied treatments (Figure 9B, Colon26, 72 h). RAD51 expression in HT29 cells at 24 h did not exceed 2-fold change and thus admitted inconsiderable (Figure 9B, HT29 cells, 24 h). After a longer incubation, the relative amount of RAD51 mRNA in all HT29 samples exposed to NPs was significantly downregulated (Figure 9B, HT29 cells, 72 h).

The protein p53 is a key tumour suppressor that has a diverse range of functions including DNA repair, regulation of cell cycle checkpoints, apoptosis, maintenance of genomic integrity, senescence and control of angiogenesis. The protein p53 signalling is one of the important intracellular signal transduction pathways that is usually dysregulated in CRC. Reports point out that TP53 mutations contribute to the aggressive and metastatic features of CRC and have prognostic and predictive significance [78,79]. After DNA damage, the amount of p53 in cells increases through posttranscriptional mechanisms, and its transactivation activity is increased, leading to the activation of downstream genes [80]. Therefore, we investigated whether exposure to NPs and NIR resulted in alterations of TP53 mRNA content in Colon26 and HT29 cells as a result of DNA damage (Figure 9C). Our results showed that a considerable, 3-fold upregulation of the TP53 gene has occurred only in NIR irradiated Colon26 cells at 24 h. Exposure to NPs, irrespective of the “NIR off” and “NIR on” or the cultivation period did not affect TP52 expression in comparison to the control group (Figure 9C, Colon26, 24 h and 72 h). In HT29 cells, regardless of the NPs treatment, the levels of TP53 mRNA resembled that in control cells at 24 h and were reduced about 5-fold in 72 h cultured cells (Figure 9C, HT29 cells).

Since there was not a clear correlation of TP53 transcription level and the observed DNA damage in Colon26 and HT29 cells after GOs and NIR treatment, the number of functional p53, in this case, was probably regulated post-transcriptionally and post-translationally, e.g., the activation of p53 through phosphorylation by protein kinases [80].

The Bcl-2-binding component 3 also known as p53 upregulated modulator of apoptosis (PUMA) is encoded by the BBC3 gene. As a member of the Bcl-2 family, PUMA can induce apoptosis through the mitochondrial pathway upon p53 activation [68]. There is an observed reduction in the p53 apoptotic response, through PUMA expression inhibition. It is thought that PUMA acts via the cytochrome c/Apaf-1-dependent pathway in regulating the p53-induced cell death [81]. In addition, PUMA could act as a pro-apoptotic factor through p53-independent signalling pathways [82]. Because of its pro-apoptotic role, this gene is a potential drug target for cancer therapy. PUMA expression is downregulated in colorectal carcinoma and has a negative correlation with the incidence of this type of cancer [68]. In our experiments, we studied the expression levels of BCC3 mRNA. Results are given in Figure 9D. We found that only incubation with GO for 24 h had some effect on PUMA mRNA expression in Colon26 cells, a two-fold increase in the BCC3 transcript was detected in comparison to the untreated control sample (Figure 9D, Colon26, 24 h). In HT29 cells, the relative concentration of BCC3 mRNA was upregulated by 2-fold upon exposure to GO–PEG NIR at 24 h. Other treatments did not influence significantly the expression of the BBC3 gene nor at 24 h neither at 72 h.

Following the logic of our experiments, we tested the levels of expression of mRNA, coding for the p21 cyclin-dependent kinase inhibitor 1A (CDKN1A), whose expression is regulated by the tumour suppressor protein p53, and participates in the p53-dependent cell cycle G1 phase arrest as a consequence of different stress stimuli [83]. The encoded protein p21 (WAF1/CIP1) binds to and inhibits the activity of cyclin-cyclin-dependent kinase 2 or -cyclin-dependent kinase4 (cyclin-CDK) complexes, and thus functions as a regulator of cell cycle progression at G1 [84]. p21 protein can interact with proliferating cell nuclear antigen PCNA, a DNA polymerase accessory factor, and plays a regulatory role in S phase DNA replication and DNA damage repair. In addition, p21 was reported to be specifically cleaved by CASP3-like caspases, which thus leads to a dramatic activation of cyclin-dependent kinase2 (cyclin A-Cdk2 complex), and may be instrumental in the execution of apoptosis following caspase activation [85]. Experiments using mice as a model organism have shown that the lack of CDK1A leads to tissue regeneration [86]. Studies of p53-dependent cell cycle arrest in response to DNA damage identified that p21 is the primary mediator of downstream cell cycle arrest. Despite the increase in cell proliferation, there is an observed increase in the p21 level of both metastases and primary tumours of the metastatic canine mammary tumours [87]. Chen and co uncovered that similarly to other growth-promoting oncoproteins, p21 also displayed an “antagonistic duality” as both overexpression (p21+) and silencing (p21−) of p21 had a pro-apoptotic effect in the absence of UVB irradiation and although apoptosis increased in all irradiated groups (control, p21+ and p21−) compared to non-irradiated ones, only p21 overexpression conferred an anti-apoptotic effect in irradiated cells [88].

Under a subset of examined experimental conditions, we found extremely high upregulation of CDKN1A gene transcription in Colon26 cells at 24 h. As shown in Figure 9E, Colon26, 24 h, the relative p21 expression increased with about 850-fold in NIR irradiated cells, 450-fold in GO plus NIR treated cells and 1100-fold in GO–PEG treated cells—in comparison to the control group. Exposure to GO–PEG NIR brought an about 4-fold decrease in p21 transcripts content relative to the untreated control. At 72 h no significant change was detected and treated cells displayed an expression rate similar to that of the untreated cells (Figure 9E, Colon26, 72 h). In HT29 cells, CDKN1A mRNA levels increased across all experimental groups incubated with NPs, about 6-fold for GO and GO NIR and approximately 8-fold for GO–PEG and GO–PEG NIR treated cells. At 72 h, again an elevated transcription of CDKN1A was observed in GO, GO–PEG and GO–PEG NIR treated cells (Figure 9E, HT29 cells). The detected increased levels of p21 mRNA in HT29 and especially in Colon26cells are following the results of other authors that link highly invasive cancer cells with increased levels of p21 [89]. Some authors indicate that p21 is an oncogenic factor in a p53-deficient environment, thus highlighting its role in tumorigenesis [90], which is exactly the case with the highly invasive Colon26 cells that are under our treatment conditions demonstrated undetectable changes in p53 mRNA levels.

The obtained results showed that the two colorectal cancer cell lines responded to the different treatments specifically concerning the expression of the studied genes. For example, NIR irradiation enhanced the transcription of ATM, RAD51, TP53 and CDKN1A at 24 h and did not affect the expression of any of the studied genes at 72 h. However, in HT29 cells, only the ATM mRNA level was increased at 24 h of treatment while again no change was detected in the five examined genes at the 72 h-time point. NIR light is proposed and used for therapy of different conditions (skin, neurological etc.) [91] and it is vitally important to know what the NIR radiation itself cause at the cellular level, e.g., on gene expression. It is worthy to note that according to our results, NIR itself changes the expression of several key cell fate signalling genes, e.g., those involved in ATM-TP53-p21 signalling pathway, as well as RAD51 in rapidly proliferating Colon26 cancer cells and the effect of NIR on gene transcription, seemed to be limited to 24 h, as at 72 h no significant alteration of mRNA quantity was observed as a consequence of irradiation with near-infrared light/NIR laser lighting.

The analysis of the results obtained for ATM and RAD51 gene expression levels showed differential transcription regulation in the two cell lines. Upon all treatments with NPs, in HT29 cells the ATM was upregulated (up to 40-fold) at 24 h and downregulated (up to 3,7-fold) at 72 h while in Colon26 cells ATM expression remained unaffected. RAD51 was upregulated (up to 27-fold) in Colon26 at 24 h but non changed at 72 h while in HT29 RAD51 transcript levels decreased at 72 h but kept control levels at 24 h. The expression of TP53 was downregulated (up to 5,7-fold) in HT29 only at 72 h and was not influenced by NPs treatments in Colon26. Under our study, the obtained results for p53 did not correspond to the observed DNA damage in Colon26 and HT29 cells after GOs and NIR treatment suggesting a posttranscriptional regulation of DNA damage response pathway by phosphorylation of p53 protein. In almost all experimental groups, BBC3 gene transcription remained at the control level that followed the steady-state expression of the upstream regulator gene TP53. Treatment of HT29 cells with NPs for 24 h or 72 h resulted in CDKN1A upregulation of about 7- and 2,6-fold respectively while in Colon26 cells only exposure to GO NIR and GO–PEG at 24 h increased the expression of this gene. A functional link between RAD51 and p21 was reported suggesting that repair of induced DNA damage may be mediated through p21 (Waf1/Cip1) and caspase-3 dependent regulation of RAD51 [92,93].

Gene expression analyses revealed that GO–PEG and GO–PEG NIR affected the regulation of the five examined genes (ATM, RAD51, TP53, BBC3 and CDKN1A) similarly (up- or down-regulation) and to a similar extent as did GO and GO NIR treatments in the two studied CRC cell lines, Colon26 and HT29. From this point of view, it is not expected the modified GO–PEG NPs alone or in combination with NIR to exert greater toxicity and poorer biocompatibility than the pristine GO nanoparticles.

## 4. Conclusions

We observed that the PEGylation of GO nanoparticles has well-pronounced biocompatibility toward colorectal carcinoma cells, besides their different malignant potential and treatment times. This biocompatibility is potentiated when GO–PEG treatment is combined with NIR irradiation, especially for cells treated for 24 h. The tested bioactivity of GO–PEG in combination with NIR irradiation induced little to no damages in DNA and did not influence the mitochondrial activity. Little changes in the cell cycle were detected. Moreover, we demonstrated that the expression levels of certain stress-responsive genes in both colorectal cancer cell lines (HT29 and Colon26) after 24 and 72 h exposure to PEGylated GO or pristine GO NPs with or without NIR irradiation for 15 min were similar. We proved that PEGylation of GO and its combination with NIR reduced the cyto-, geno- and mitotoxicity of these nanoparticles. These findings highlight the possibility of the as-modified NPs to be used as smart nanocarrier of antitumor drugs in future combined chemo–photo therapies of colon cancer. We further demonstrated that the synergistic effect of GO–PEG with NIR depends on the invasive potential of colon cancer cells.

## Figures and Tables

**Table 1:** Primers used in RT-qPCR reactions. For all studied genes two sets of primers pairs were used: For the mouse and human genes. The human genes primers are colored in gray.

Name	Sequence 5′–3′
Hs_GAPDH_For	ACCAGGTGGTCTCCTCTGACTTCAA
Hs_GAPDH_Rev	ACCCTGTTGCTGTAGCCAAATTCG
Mmus_GAPDH_For	CGACTTCAACAGCAACTCCCA
Mmus_GAPDH_Rev	AGCCGTATTCATTGTCATACCAGG
Hs_ATM_For	TGCTGTGAGAAAACCATGGAAGTGA
Hs_ATM_Rev	TCCGGCCTCTGCTGTAAATACAAAG
Mmus_ATM_For	AGGTGTCTTCAGAAGGTGCTGTG
Mmus_ATM_Rev	CCTCTACAATGGTCAGCAGGGT
Hs_TP53_For	AACAGCTTTGAGGTGCGTGTTTGTG
Hs_TP53_Rev	AGAGGAGCTGGTGTTGTTGGGCA
Mmus_TP53_For	GGAGAGTATTTCACCCTCAAGATCC
Mmus_TP53_Rev	AGACTCCTCTGTAGCATGGGC
HsBBC3_For (PUMA)	TACGAGCGGCGGAGACAAG
HsBBC3_Rev (PUMA)	GGTAAGGGCAGGAGTCCCAT
Mmus_BBC3_For (PUMA)	TACGAGCGGCGGAGACAA
Mmus_BBC3_Rev (PUMA)	GCTCCAGGATCCCTGGGTAA
Hs_CDKN1a_For	AGAGGAAGACCATGTGGACCTGTCA
Hs_CDKN1a_Rev	AGAAATCTGTCATGCTGGTCTGCC
Mmus_CDKN1a_For	ATCTCAGGGCCGAAAACGGA
Mmus_CDKN1a_Rev	TCTTGCAGAAGACCAATCTGCG
Hs_Rad51_For	TCAAGCATCAGCCATGATGGTAGAA
Hs_Rad51_Rev	AGAAACCTGGCCAAGTGCATCTG
Mmus_Rad51_For	CCCAAGTAGATGGAGCAGCCA
Mmus_Rad51_Rev	TTTCTCAGGTACAGCCTGGTGG
